# Epidemiologic and economic considerations regarding persistently infected cattle during vaccinate-to-live strategies for control of foot-and-mouth disease in FMD-free regions

**DOI:** 10.3389/fvets.2022.1026592

**Published:** 2022-10-20

**Authors:** Shankar Yadav, Amy H. Delgado, Amy D. Hagerman, Miranda R. Bertram, Karla I. Moreno-Torres, Carolina Stenfeldt, Lindsey Holmstrom, Jonathan Arzt

**Affiliations:** ^1^Foreign Animal Disease Research Unit, Plum Island Animal Disease Center, Agricultural Research Service, United States Department of Agriculture, Greenport, NY, United States; ^2^Center for Epidemiology and Animal Health, Animal and Plant Health Inspection Service, United States Department of Agriculture, Fort Collins, CO, United States; ^3^Plum Island Animal Disease Center Research Participation Program, Oak Ridge Institute for Science and Education, Oak Ridge, TN, United States; ^4^Department of Agricultural Economics, Oklahoma State University, Stillwater, OK, United States; ^5^Department of Diagnostic Medicine/Pathobiology, Kansas State University, Manhattan, KS, United States

**Keywords:** foot-and-mouth disease, FMD, vaccination, carrier, virus, economic model, simulation model, persistent

## Abstract

Development of a foot-and-mouth disease (FMD) carrier state following FMD virus (FMDV) infection is a well-established phenomenon in cattle. However, the proportion of cattle likely to become carriers and the duration of the carrier state at a herd or population-level are incompletely understood. The objective of this study was to examine the epidemiologic and economic impacts of vaccination-to-live strategy in a disease-free region or country. We developed and simulated scenarios of FMD spread and control in the US livestock population, which included depopulation for a limited period, followed by a vaccinate-to-live strategy with strong biosecurity and movement restrictions. Six scenarios of FMD spread and control were simulated in the InterSpread Plus (ISP) modeling tool. Data on the number of infected and depopulated cattle (by operation types) from ISP model runs were used to estimate the monthly number of infected but not depopulated (potential carrier) cattle after the infection. Using available literature data on the FMD carrier state, we estimated the monthly proportion of carrier cattle (from infected but not depopulated cattle) over time following infection. Among the simulated scenarios, the median (25th, 75th percentile) number of infected cattle ranged from 43,217 (42,819, 55,274) head to 148,907 (75,819, 205,350) head, and the epidemic duration ranged from 20 (11, 30) to 76 (38, 136) days. In general, larger outbreaks occurred when depopulation was carried out through longer periods, and the onset of the vaccination was late (*p* > 0.05). The estimated proportion of surviving cattle, which were infected and not depopulated and had the potential to become persistently infected ranged from 14 to 35% of total infected cattle. Production losses in beef and dairy sectors were higher when outbreaks started in multiple states simultaneously, but production losses were small compared to trade losses and consumer avoidance losses. These results can be used to inform the consideration of a vaccinate-to-live strategy for FMD outbreaks and the development of appropriate post-outbreak management strategies. Furthermore, this output will enable a more detailed examination of the epidemiologic and economic implications of allowing convalescent cattle to survive and remain in production chains after FMD outbreaks in FMD-free regions.

## Introduction

Challenges in foot-and-mouth disease (FMD) control are numerous and complex. While some of these are social and/or political, many are directly linked to the inherent characteristics of the virus—extreme contagiousness, a wide range of affected hosts, and multiple viral serotypes that do not confer cross-protection (1–4). As a result, the introduction of FMD into a previously free region requires a rapid response, which synergistically addresses all of these factors while considering the practical and logistical aspects of emergency response and the concurrent economic impacts. Given the intricacies of FMD outbreak responses in the United States, both consumers and livestock producers are increasingly expecting that every tool, including vaccination, will be considered in the event of an outbreak.

Vaccination has been an important tool in controlling and eradicating FMD, particularly in endemic settings, but also during outbreaks in previously free countries (1, 5, 6). Vaccination offers several benefits to an emergency response, including reducing the need for large-scale depopulation of animals and the environmental impact associated with the disposal of depopulated animals. However, vaccination as a response tool in a previously FMD-free country also brings challenges, particularly related to decisions on how to manage vaccinated animals. Under the US Department of Agriculture's (USDA) FMD response plan, the management of vaccinated animals varies across four different response strategies —emergency vaccination to kill, emergency vaccination to slaughter, emergency vaccination to live, and emergency vaccination to live without stamping out of infected animals or vaccinates (7). In geographically constrained outbreaks where the disease can be controlled and eradicated in a short amount of time, there are few economic incentives for a vaccinate-to-live strategy. Guidelines suggest trade embargos for six months when the vaccinated animals are allowed to live versus three months when the vaccinated animals are culled. In addition, there are additional resources required to manage vaccinates in a vaccinate-to-live strategy including costs and effort required for testing and tracking vaccinated animals through their productive life spans (8–10).

However, there are benefits to vaccinate-to-live approaches. These alternative approaches to vaccinate-to-kill and stamping-out can reduce the costs of on-farm responses, such as depopulation, carcass disposal, and indemnity paid to producers for animals taken during the response. In addition, genetically valuable animals may be preserved, and disruptions in production chains could be reduced. Lastly, there are scenarios in which vaccinate-to-live is unavoidable, most notably when the number of vaccinated animals exceeds the practical capacity for depopulation. In addition, for countries that are not highly dependent on livestock and meat export markets, the economic advantage associated with reduced trade embargoes of vaccinate-to-kill, may be overcome by the value of protein saved due to reduced depopulation. However, the potential use of vaccinate-to-live is confounded by some of the biological properties of the virus, particularly the carrier state (11).

Foot-and-mouth disease virus (FMDV) is highly transmissible, and while vaccination can substantially reduce transmission and the development of clinical signs, vaccinated animals exposed to the virus may still become infected and develop antibodies (12). In addition, regardless of vaccination status or virus strain, a substantial proportion (≥50% of infected cattle) of infected ruminants will develop a subclinical persistent phase of infection (1, 11). This carrier state is identified by detection of infectious virus (FMDV) in oropharyngeal fluid (OPF) more than 28 days post-infection (dpi) (12, 13). Most genetic diversity of FMDV (14) develops during the carrier state (15); however, the epidemiologic significance of carriers in relation to disease transmission remains unclear (11).

Concerns about the risks posed by FMDV carriers remain due to historical accounts of FMD outbreaks believed to have resulted from transmission between persistently infected carriers and susceptible animals. Although published experimental studies have almost uniformly reported a lack of transmission from carriers to contact exposed animals (16, 17), some studies have demonstrated that OPF from carriers is capable of infecting naïve cattle (18). The duration of the carrier state is likely influenced by numerous factors, as evidenced by the diversity of reported lengths in the literature (19–22). For example, the carrier state has been shown to last for up to 4–5 years in African buffalo (*Syncerus caffer*), 4 months to 3.5 years in cattle (19, 20), and up to 9 months in sheep (21–24). The discrepancies in the duration of the carrier state may be due to differences in virus (serotype, strain or virulence), host (genetic differences, nutritional or immunological status), environment (climate, mineral intake), and methods of virus detection across studies (14).

Despite the limited epidemiologic evidence, carriers have been recognized as a barrier to re-establishing disease freedom following an outbreak (8, 25). Proving disease freedom is a critical step to the gradual or immediate resumption of international trade. As a result, when vaccination is used as part of the outbreak response strategy, vaccinated herds must be monitored and managed to reduce the risk associated with the presence of infectious virus. With increasing expectations from livestock producers and the general public regarding the use of vaccination, additional information on the duration and relevance of the carrier state is needed in order to design monitoring plans for vaccinated herds, particularly for large or complex livestock operations. While some work has been done to model the prevalence of carriers after reactive vaccination, recent studies offer new insights into the prevalence and duration of the carrier state (19, 26).

The objective of this study was to examine the epidemiologic and economic impacts of alternative approaches for FMD control that limit the use of depopulation, while taking into account the presence of carrier cattle over time in the affected population. To achieve this, we developed and simulated scenarios of FMD spread and control in the US livestock population, which included depopulation for a limited period, followed by a vaccinate-to-live strategy with strong biosecurity and movement restrictions. Based on currently available data from the literature, we estimated the prevalence and duration of the carrier state over time for varied cattle production systems. The resulting scenarios were also used to estimate the economic consequences of deviating from a stamping-out strategy, including production losses in infected herds, market impacts, and response costs. Many questions remain about the social and trade ramifications of managing carrier and vaccinated animals.

## Materials and methods

### Study population

The FMD model scenarios developed in this study were based on the national livestock population of the United States. The farm data was obtained using a micro-simulation model called the Farm Location and Animal Population Simulator (FLAPS), which generates synthetic farm populations with production types, herd sizes, and geographical coordinates, while taking into account livestock census data (27). To account for the geographical diversity in demographics of livestock operations, animals, and their movement, the mainland of the United States was divided into five discrete regions: Pacific (PC), Midwest (MW), Great lakes (GL), North East (NE) and South East (SE). Altogether 1.82 million livestock operations, which included bison (0.14%), cattle (84%), goats (7%), sheep (3%), and pigs (0.2%) were included in the model. Operation characteristics, including movement destinations and frequencies, varied based on the herd size (Table 1 and Supplementary Table S1). Cattle farms were categorized into four different production types based on the age and management practices of animals—cow calf, stocker, dairy, and feedlot (Supplementary Table S1). Among cattle farms in the population dataset, 2% were classified as large farms (≥200 head for cow calf, ≥500 head for dairy, and ≥1,000 head for feedlot). The stocker farms were identified as small operations. The cow calf (small: 96%, large 4%), dairy (small: 95%, large: 5%), feedlot (small: 93%, large: 7%), and stockers (all small) constituted 47, 4, 2, and 47% of the total cattle farms in the population file.

**Table 1:** Descriptive statistics of foot-and-mouth disease susceptible livestock population incorporated in the InterSpread Plus (ISP) model for the simulation of model scenarios.

**Farm type**	**Operation type**	**Number of farms**		**Median (5th, 95th) herd size**	
		**Small**	**Large**	**Small**	**Large**
Cattle	Cow calf	700,655	25,980	14 (2, 103)	293 (210, 1,038)
	Dairy	60,715	3,342	40 (1, 231)	1,257 (575, 4,071)
	Feedlot	24,813	1,772	59 (12, 430)	2511 (1,072, 25,031)
	Stockers	717,330		9 (2, 161)	
Bison	Bison	2,547		10 (1, 223)	
Goat	Goat	127,954		11 (6,58)	
Sheep	Sheep	87,935		13 (3, 148)	
Swine	Small swine enterprises	47,062		6 (1, 45)	
	Farrow to feeder	353	119	193 (105, 765)	3,921 (1,387, 28,739)
	Farrow to finish	2,234	1,480	289 (110, 802)	4,291 (1,331, 15,152)
	Farrow to wean	322	762	309 (106, 848)	4,501 (1,283, 17,126)
	Grower to finisher	2,134	6,063	466 (134, 850)	4,373 (1,394, 13,760)
	Nursery	203	1,030	595 (160, 880)	4,638 (1424, 14,219)
	Others	271	945	320 (107, 815)	4,362 (1,411, 13,472)
Dealer	Dealer	3,427		56 (5, 114)	

Among the pig farms, 83% were small and 17% were large farms. There were seven different operation types included in the population file: small swine enterprises (75%), farrow to feeder (1%), farrow to finish (6%), farrow to wean (2%), grow to finisher (13%), nursery (2%), and others (2%) (Table 1). Large operations were considered to be those with ≥1,000 head for all production types (Supplementary Table S1). The small swine enterprises operations were backyard and hobby swine farms, with < 100 heads. Bison, goat, and sheep operations were recognized as small farms (Table 1 and Supplementary Table S1). Dealers were also included in the model. Dealers were considered to be small operations which could have different species present that represented frequent aggregation and dispersion points outside of normal livestock markets.

### Epidemic model description

#### Disease spread

InterSpread Plus (ISP) *version 6.0* model software was used to simulate the between-herd spread of FMD in the US livestock population (28). ISP is a state-transition, stochastic and spatial modeling tool for the simulation of FMD and other similar diseases (29). The unit of interest in the model was individual livestock operations. FMD epidemics were either simulated from a single farm or two farms depending on the model scenarios. The simulation proceeded by the time-step of 1 day. The herd-level disease parameters (such as incubation phase duration and maximum infectiousness) were assigned stochastically in the models specific to animal species. The herd-level incubation phase durations (in days) assigned were Poisson (3.8) for bison (30), Poisson (5.9) for cattle (31), Poisson (6.59) for goat and sheep (30), and Poisson (5.58) for pigs (32). The infectious phase duration was assigned to be [Triangular (30, 34, 42)] days. Model parameters were based on transmission characteristics of FMDV serotype O, and all outbreaks were assumed to start in January, for the purposes of livestock placement. Depending on the model scenarios, disease spread initiated within a single state (California or Texas) from a single farm, or within two states simultaneously (California and Texas) from two farms (Table 2).

**Table 2:** Descriptions of the foot-and-mouth disease model scenarios.

**Characteristics**	**Scenarios**					
	**1**	**2**	**3**	**4**	**5**	**6**
Index herd location	CA	TX	CA & TX	CA	TX	CA & TX
Index herd type	Large dairy	Large feedlot	Large dairy & large feedlot	Large dairy	Large feedlot	Large dairy & large feed lot
Index herd size	3,596	42,806	3,596 & 42,806	3,596	42,806	3,596 & 42,806
Onset of depopulation	Day 14					
Depopulation duration	28 days			14 days		
End of depopulation	Day 42			Day 28		
Onset of vaccination	Day 43			Day 29		

CA: State of California, TX: State of Texas. In scenarios 3 and 6, the epidemic was initiated on day 0 from a farm in CA and 7 days later, another farm in TX was modeled to infect.

Transmission of FMDV between farms could occur through multiple routes, including direct contacts such as animal movements, indirect contacts, such as shared vehicles or personnel, airborne transmission, and local area spread at short distances. Movement frequencies, distances, and destination types were unique to each production type, and these varied for movements to markets (Supplementary Table S2), and by the region of farm location and size (small vs. large) (Table 3, Supplementary Tables S3–S6). The daily probability of transmission of FMDV after contact between an infected and susceptible farm was estimated based on the hypergeometric probability of shipping at least one infected animal off of an infected farm given the average herd size, shipment size, and the number of infected animals in a herd on a given day. This parameter was estimated and assigned for each of the animal species (bison, cattle, goat, sheep, pig), dealers, and markets (Supplementary Figure S1).

**Table 3:** The distributions of the average frequency of direct contact movements originated from respective farm types and operations in the various regions of the United States.

**Farm/operation**	**Frequency per day**
Bison	Poisson (0.0021)
Cow calf (large) in MW & PC region	Poisson (0.009)
Cow calf (large) in another region	Poisson (0.005)
Cow calf (small) in MW& PC region	Poisson (0.004)
Cow calf (small) in another region	Poisson (0.002)
Dairy (large)	Poisson (0.0986)
Dairy (small)	Poisson (0.0356)
Dealer	Poisson (0.1471)
Feedlot (large)	Poisson (0.03)
Feedlot (small)	Poisson (0.03)
Goat	Poisson (0.0022)
Sheep	Poisson (0.0026)
Stockers	Poisson (0.007)
Small swine enterprises	Poisson (0.0023)
Swine farrow to feeder	Poisson (0.1049)
Swine farrow to finish	Poisson (0.0209)
Swine nursery	Poisson (0.0868)
Swine farrow to wean	Poisson (0.4068)
Swine grow to finish	Poisson (0.0015)
Swine other	Poisson (0.0413)

MW, Mid-West; PC, Pacific region of the United States.

Indirect contacts between infected and susceptible farms included connections such as shared farm workers, veterinarians, vehicles, equipment. Indirect contacts were modeled as high or low risk based on the potential for viral contamination and contact with animals. High-risk indirect contacts included veterinarians, customers, dealers, employees with livestock at residence, extension agents, livestock haulers including those used for dead box pick-ups, and manure haulers. Low risk indirect contacts included commodity/feed trucks, shared equipment, drivers of livestock haulers, nutritionist, feed company consultants, other vehicles such as postal deliveries, and visitors. The indirect contact rates assigned in the ISP model scenarios are summarized in Table 4. The probability that an indirect movement occurs within a certain distance varied by production type, with most movements occurring within 20 km of the original farm (Supplementary Table S7). The probability that infection occurs was estimated for high risk and low risk indirect contact movements separately, and we assumed that low risk indirect contacts followed basic biosecurity protocols, leading to a reduced risk of disease transmission (Supplementary Figure S2).

**Table 4:** Distributions of indirect contact rates among the livestock farms in the United States used to incorporate in the InterSpread Plus model.

**Operations**	**Pacific region**		**Other regions**	
	**High risk**	**Low risk**	**High risk**	**Low risk**
Bison	Poisson (1.9167)	Poisson (0.5747)	Poisson (1.9617)	Poisson (0.5747)
Cow calf (large)	Poisson (0.11)	Poisson (0.2244)	Poisson (0.11)	Poisson (0.2244)
Cow calf (small)	Poisson (0.053)	Poisson (0.098)	Poisson (0.053)	Poisson (0.098)
Dairy (large)	Poisson (1.5873)	Poisson (0.3891)	Poisson (1.5873)	Poisson (0.3891)
Dairy (small)	Poisson (0.4596)	Poisson (0.1252)	Poisson (0.4596)	Poisson (0.1252)
Dealer	Poisson (0.147)	Poisson (0.164)	Poisson (0.147)	Poisson (0.164)
Feedlot (large)	Poisson (1.48)	Poisson (6.46)	Poisson (1.48)	Poisson (6.46)
Feedlot (small)	Poisson (0.15)	Poisson (0.28)	Poisson (0.15)	Poisson (0.28)
Stockers	Poisson (0.006)	Poisson (0.017)	Poisson (0.006)	Poisson (0.017)
Goats	Poisson (0.335)	Poisson (0.0452)	Poisson (0.335)	Poisson (0.0452)
Sheep	Poisson (01961)	Poisson (0.0428)	Poisson (0.1961)	Poisson (0.0428)
Small swine enterprises	Poisson (0.002)	Poisson (0.0940)	Poisson (0.002)	Poisson (0.0940)
Commercial swine (large)	Poisson (2.214)	Poisson (1.3053)	Poisson (2.214)	Poisson (1.239)
Commercial swine (small)	Poisson (0.3486)	Poisson (0.3402)	Poisson (0.3387)	Poisson (0.2119)

Local area spread was assumed to occur at short distances (within 4 km of an infected farm) through insects, rodents, or other unknown factors. The probability of disease transmission due to local spread was modeled separately based on the status of the infected farms. Undetected, infected farms were given highest risk for disease transmission in compared to detected but not depopulated farms, or depopulated farms which still needed to complete carcass disposal (Supplementary Figures S3A–C). The airborne spread of FMDV was assumed to occur within 10 km of infected swine farms after the onset of clinical signs, with the probability of transmission declining over distance (Supplementary Figure S3D).

#### Control measures

Initial detection was fixed on day 11 for all scenarios and after that it was based on background passive surveillance. The probability of detection during passive surveillance varied by days post-onset of clinical signs and species affected, with swine having the highest probability of detection and dealers having the lowest (Supplementary Figure S4). Following detection of an infected farm, control measures were initiated on day 1 after the detection, including the establishment of control zones. Two types of radial control zones (inner and outer) were included in the model. The inner control zone was from 0 to 10 km of the infected farms, and the outer control zone was from 10 to 20 km away from the infected farms. After the first detection, direct contact tracing, indirect contact tracing, and surveillance of all farms within the 10 km zone of the detected farms were initiated. Movement restrictions were imposed on all farms within inner control zones immediately after detection. The percentage of animal movements restricted ranged from 60% for swine up to 85% for cattle, while only 25% of indirect contact movements were restricted.

In all scenarios, depopulation of infected animals was initiated following detection on day 14; however, the duration of depopulation efforts varied between 14 and 28 days (Table 2). While initial depopulation capacity was assumed to be small (4 farms/day for the first 2 days of control activities), depopulation capacity ramped up quickly and varied by operation type. From day 3 to the end of the depopulation effort, the assigned depopulation capacity distributions were Betapert I4, 6, 10) for large cattle farms, Betapert (2, 4, 6) for small cattle, goat and sheep farms, Betapert (4, 8, 28) for large feedlots, Betapert (1, 2, 4) for large swine farms, and Betapert (2, 2, 6) for small swine farms. After 14 or 28 days of depopulation efforts, all depopulation was ceased and vaccination was initiated at day 29 or 43 post-introduction, respectively (Table 2). All cattle, bison, and swine in the 10 km zone of the detected farms were vaccinated at the rate of 85,000 cattle, 1,000 bison, and 14,000 pigs per day. Sheep and goats were not vaccinated in these scenarios. All vaccinated cattle, bison, and pigs were assumed to live out their normal production periods.

### FMD model scenarios

Based on the previously described model structure, six different model scenarios were simulated (Table 2). Briefly, the model scenarios were run from either one-farm (scenarios 1, 2, 4, and 5) or two-farms (scenarios 3 and 6). The characteristics of index farms differed in herd size, location, and operation types. The detected farms in a scenario were depopulated for either for 28 days (scenario 1, 2, 3) or 14 days (scenario 4, 5, 6). Vaccination was initiated after the cessation of depopulation activities, i.e., at 43rd day in scenario 1, 2, and 3 and 29th day in scenario 4, 5, and 6. A shorter duration of depopulation in the model scenarios was designed intentionally to allow vaccination-to-live strategy and thereby to estimate the risks and challenges due to emergence of persistence infection in cattle of the US livestock population. The models were simulated for 200 iterations; each iteration was simulated for 730 days (maximum). The major outcomes of the ISP model were to estimate the epidemic duration, epidemic size, number of infected and depopulated farms and animals, and the number of infected cattle potentials for the emergence of the persistent infection. To test for significance among model scenarios we used the Kruskal-Wallis test with Bonferroni correction for multiple comparison.

### Estimation of persistently infected cattle

In this study, we quantified the number of persistently infected cattle and extinction of persistent infection over time after infection. First, the numbers of infected but not depopulated cattle (all cattle, cow calf, dairy, and feedlot and stockers combined) were estimated for each month after onset of infection using the infection data and depopulation data from ISP model outcomes for each of the scenarios. These were the monthly numbers of cattle with potential for persistent infection. Second, using literature data (Table 5), an equation (y = 0.59–0.021x) was derived to estimate prevalence of persistently infected cattle over the succeeding month after the infection. In the equation, y is the prevalence of the persistent infection and x is the month after infection. The equation demonstrated that the prevalence of persistently infected cattle was 57% after the first month of infection (28 days post infection), 55% in the second month (56 days post infection), and consequently the persistent infections were cleared by 29 months post-infection. This equation was used to estimate the numbers of persistently infected cattle at a month after FMD infection. For example, in scenario 1, altogether 28,505 cattle remained that had been infected and not depopulated; these are the cattle with potential of establishing persistent infection after 28 days of infection. Using the equation, it was estimated that after the first month of infection, 57% of these cattle (16,248) were persistently infected. When these cattle reached the third month post infection, 55% (15,678) remained persistently infected indicating that 570 cattle (2%) had cleared the persistent infection within this period. Consequently, all of the persistently infected cattle in this scenario had cleared infection by 29 months post onset of infection.

**Table 5:** Published studies from which data on the prevalence of persistently infected cattle were extracted to derive an equation.

**References**	**Prevalence**	**Months after outbreak**	**Species**
de Carvalho Ferreira et al. (33)	10.8%	12 months	Cattle and buffalo
Hayer et al. (34)	38%	7.5 months	Dairy cattle
	14%	10.5 months	Dairy cattle
Hayer et al. (20)	67%	6 months	Dairy catt
	55%	14 months	Dairy cattle
	51%	11 months	Dairy cattle
Tenzin et al. (17)	62%	28 days	Cattle
	52%	7 months	Cattle
Hedger (35)	20%	7 months	Cattle

### ISP model outcomes inputs for economic model

In order to facilitate comparison of economic impact results to epidemiologic outcomes, an index was created based on ranking iterations by key outcomes from the ISP model within each scenario. The index was comprised of an ordinal ranking herds infected, head infected, duration, and states affected, and creating an index for each iteration based on equal weighting of each epidemiological outcome. The median, 25th percentile, and 75th percentile based on this index was analyzed in the United States Partial Equilibrium Model (36). Although the economic model has many outcomes, the ones reported for this study are the change in returns to capital and management from livestock and agricultural product sales, the change in returns to dairy cattle and milk, and the change in returns to beef cattle and beef. In each case, the scenario specific values for each quarter are subtracted from the quarterly no-disease base from 2019 to 2021.

### Cost of response and economic model

Economic impacts for animal health outbreaks were categorized as production losses, costs of disease response on farms, and market impacts. Production losses included the loss of animals available to the market due to mortality and depopulation, as well as reduced weight gain, milk production and fecundity that resulted from clinical infection. Observations of production losses in the published literature were used in the absence of observations from FMD outbreaks in the US (Table 6). Details on the production loss parameters can be found in Supplementary material.

**Table 6:** Published studies from which data on production and demand impacts in cattle were extrapolated to estimate economic impacts of an FMD vaccinate-to-live without stamping out response.

**References**	**Production loss**	**Description**	**Type of loss**
Ferreira et al. (37)	−1.4%	Reduced rate of gain during clinical infection. Normal weight gain post- clinical infection	Average daily weight gain in beef cattle
Lyons et al. (38)	−35%	During clinical infection	Pounds of milk production in dairy cattle
	5% increase in lbs milk per month	7 month recovery period in which milk production increases steadily from the 35% loss until it is back to normal. Uniform recovery gains were assumed.	Pounds of milk production in dairy cattle
Doel (39)	−10%	Rate of abortion in pregnant cows during clinical infection	Dairy and beef cows and heifers pregnancy losses
Rufael et al. (40)	−2.8%	Death rate in calves under 2 years of age	Unweaned beef and dairy cattle
Mu et al. (41)	−0.5%	Consumer avoidance of beef, pork and lamb due to risk perception.	Beef, pork and lamb domestic consumption

The ISP disease spread results in infection by herd type, depopulation by herd type, duration of outbreak, and states with infected livestock, which were used as inputs in the economic modeling. Losses were tracked across time based on the quarter in which infection occurred for each herd in ISP results. The reduced beef supply available from fed cattle, the increased beef supply from culled dairy cows that aborted, and the reduced fluid milk supply for processing were incorporated as production shocks, along with the meat and milk removed from supply due to depopulation and calf deaths, in the US Partial Equilibrium Model by quarter (36).

Total on-farm costs of disease response included surveillance, depopulation and indemnification of depopulated animals, disposal of carcasses and potentially contaminated materials, cleaning and disinfection of facilities, and vaccination. Response costs were estimated in US dollars per head by production type. The response cost burden to producers for farm labor and equipment used to manage disease was not included, recognizing that some costs are part of normal herd management and that not all of the costs to producers can be foreseen. In addition, we recognize the existence of additional costs in an outbreak, but this study will focus solely on those costs associated with disease response activities on farms that are designated as infected, vaccinated and/or under surveillance at some point in the outbreak and recovery period. Total on-farm costs of disease response was carefully differentiated from the “total cost of the outbreak” which would include a variety of other costs to producers, agribusinesses, and the government. For example, the cost to a feed company of cleaning and disinfecting trucks making feed deliveries in surveillance zones, or the cost of state and federal animal health laboratory personnel. Thus, this estimate is limited to a taxpayer cost for on-farm response activities. Additional details on costs of on-farm response can be found in Supplementary materials document.

In addition to production losses and costs of response on farms that were directly impacted, losses may also accrue due to market responses. It is unknown how US trade partners or domestic consumers would respond to a vaccinate to live strategy, but literature and historical experiences for other diseases offer a place to begin developing market shocks for both trade embargoes and domestic consumer avoidance of animal products from susceptible species. Trade embargoes for beef, pork and limited dairy products were derived from the literature and World Animal Health Organization (OIE) trade guidelines (8). This is consistent with Schroeder et al. (42), the only other published study to compare vaccinate to live and vaccinate to kill. Domestic demand can also be affected by consumer avoidance, although these effects have been found to be relatively small in percentage terms and of short duration as in Mu et al. (41) examination of highly pathogenic avian influenza and bovine spongiform encephalopathy Based Mu et al.'s findings, a shallow, negative shock (−0.5%) was imposed to US beef and pork demand that was sustained through the outbreak. Recovery was allowed to occur quickly afterward. Although not modeled directly, there may actually be a positive perception by consumers of a vaccinate to live strategy since images of mass depopulation was associated with a negative public response in the UK in 2001 as found in Thompson 2002 (43). The impact of production losses, depopulation and death losses, trade embargoes and domestic consumption losses on markets were estimated using the United States Partial Equilibrium Model (USPEM) (36). This model is a national price-endogenous economic model that endogenously estimates changes in market prices and economic welfare in calendar quarter time steps. Production losses and demand shocks, as described above, were imposed on the model as exogenous shocks. Output includes market prices and producer welfare, which is defined as the difference between the schedule of prices at which producers are willing and able to supply a good in varying quantities supplied, and the price they actually realize in the market for those quantities supplied. It is different from profit in that producer welfare accounts for fixed, or sunk, costs of production.

## Results

### Livestock demographics

The simulation model consisted of 1.82 million livestock farms distributed across animal production types as follows: 0.14% (bison), 84% (cattle), 7% (goat), 5% (pig), and 3% (sheep). Of the 84% designated as cattle farms, cow-calf and stockers made up 47% each, while dairy and feedlot farms made up 4 and 2%, respectively. Of the 5% designated as pig farms, 75% were small swine enterprises and 13% were grower to finisher farms. The majority of cattle and pig farms (98% each) were small holdings (Table 1), and the herd size across farm types ranged from: 1 to 50,528 head (bison), 1 to 100,734 head (cattle), 1 to 4,837 head (goat), 1 to 422,475 head (pig), and 1 to 48,160 head (sheep).

### Number of infected and depopulated farms and animals

**Figure 1:**
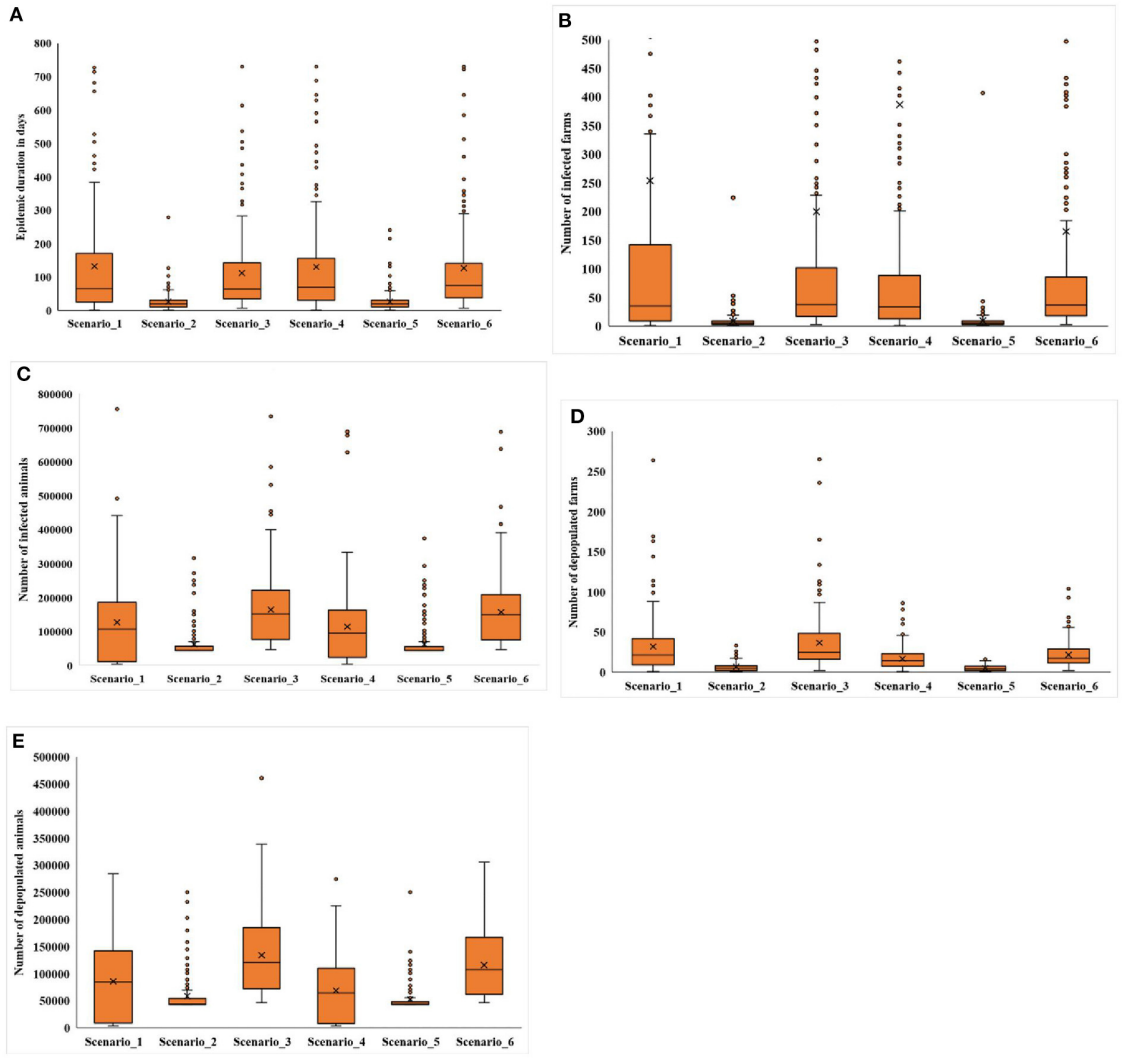
Box plots for **(A)** epidemic duration, **(B)** number of infected farms, **(C)** number of infected animals, **(D)** number of depopulated farms, and **(E)** number of depopulated animals obtained from InterSpread Plus model scenarios. The middle, lower, and the upper line of the box represents the median, 25th, and 75th percentile. The whiskers represent 1.5 times of the interquartile range. The sign × represents the mean, and the dots are the outliers detected by the analytic tool.

Among the simulated scenarios (Table 2), the median number of infected farms ranged from 5 to 38 farms, whereas the median number of infected animals ranged from 43,256 to 150,572 animals (Figure 1). Across the six scenarios, we found that the number of infected farms estimated from scenarios 2 and 5 were significantly smaller than the other scenarios (*p* < 0.0001), while the comparison among the remaining scenarios showed no significant difference in outbreak size (*p* > 0.05). Additionally, the number of infected farms was not significantly different between the 28- and 14-day depopulation strategies (*p* = 0.705). However, a slightly higher number of animals were infected in the 28-day depopulation scenarios when compared to the 14-day depopulation scenarios (*p* =0 .54).

The median number of depopulated farms among the simulated scenarios ranged from 4 to 25 farms (Figure 1), and the number of depopulated farms was significantly higher in the scenarios using the 28-day depopulation strategy (*p* < 0.05) except in scenarios 2 and 5 (*p* = 0.4538). The median number of depopulated animals among the simulated scenarios ranged from 43,162 to 120,282 animals, while the number of depopulated cattle ranged from 43,134 to 106,625 head. Like depopulated farms, a significantly higher number of animals (*p* = 0.0058) and cattle (*p* = 0.0073) were depopulated under the 28-day depopulation strategy compared to the 14-day strategy.

### Epidemic duration

The median epidemic duration ranged from 20 to 76 days among the simulated scenarios (Figure 1). The shortest epidemic duration was observed when the outbreak was initiated in a feedlot herd (scenario 2 or 5), while the longest epidemic duration occurred when infection was initiated in two herds simultaneously (scenario 6) (*p* < 0.0001). The epidemic duration was not significantly affected by the duration of depopulation (*p* = 0.4966).

### Persistently infected cattle

**Figure 2:**
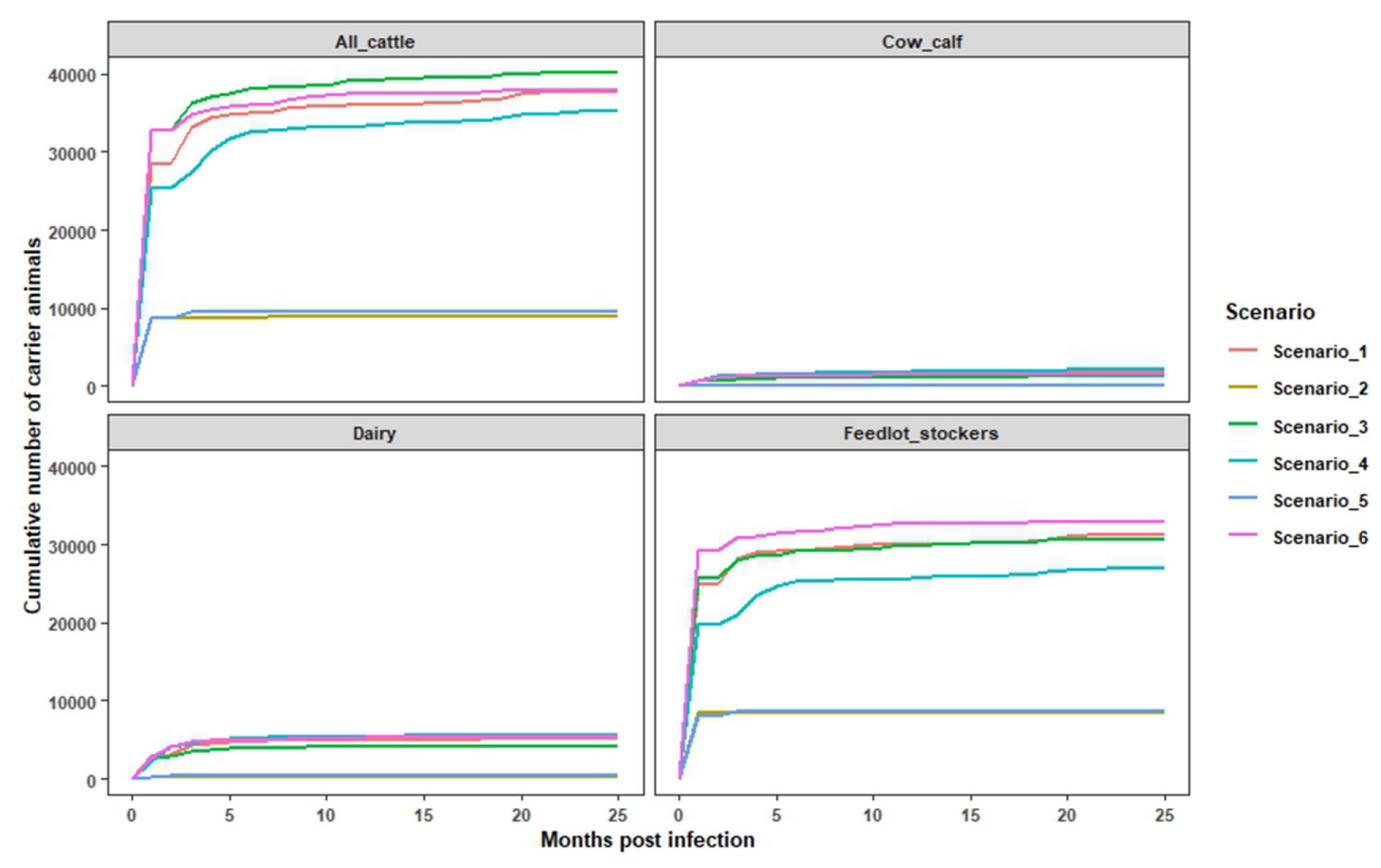
The estimated monthly cumulative numbers of infected but not depopulated cattle, corresponding to potential FMDV carriers. The estimates were obtained using the infection file and depopulation file data from InterSpread Model specific to all cattle, cow calf, dairy, and feedlot and stocker combined.

**Figure 3:**
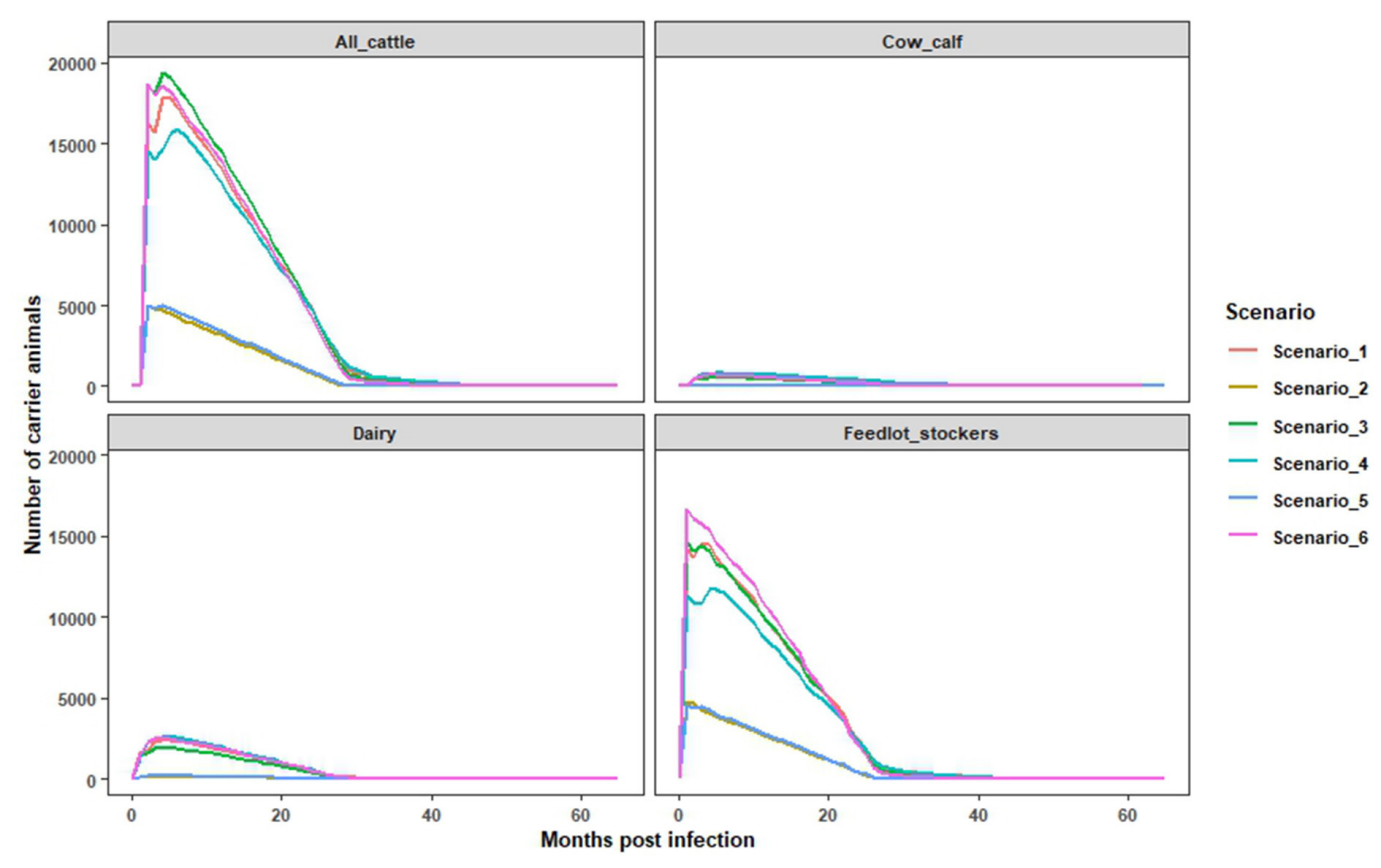
The estimated numbers of monthly persistently infected cattle and their extinction over the time. The estimates were obtained using the Microsoft Excel based model for all cattle, cow calf, dairy, feedlots and stockers (combined).

In this study, we simulated a shorter duration depopulation strategy to examine scenarios in which exposed cattle could remain in the population for the full duration of their production life. It was found that 20–38% of infected cattle were not depopulated and had the potential to progress to the FMD carrier state, which is defined as maintaining detectable virus after 28 days of infection. Amongst non-depopulated and infected cattle, 57% transitioned into the carrier state after the first month of infection with the potential to remain in the population from 30 to 52 months post infection. The monthly cumulative number of persistently infected cattle, across farm types, is a function of the total number of infected animals, and correspondingly, these values were highest in scenarios where the outbreak size was large (Scenarios 1, 3, and 6) (Figure 2). Over the epidemic period, the cumulative number of infected cow-calf, dairy, and feedlot/stockers cattle ranged from 98 to 2,266 head for cow calf; 332–5,333 head for dairies; and 8,637–32,953 head for feedlot/stocker operations. The estimated number of potentially persistently infected cattle varied across scenarios, consistent with differences in outbreak size and depopulation capacity within a farm type. For example, in scenarios 2 and 5, around 5,004 cattle were estimated to be persistently infected, compared to an estimate of 18,655 in scenarios 3 and 6, after the first month of the outbreak [Figure 3 (all cattle)]. Among the different types of cattle farms, we found that feedlots and stocker farms accounted for the highest proportion of cattle with the potential to become persistently infected, followed by dairies and cow calf operations (Figure 3). These findings are consistent with the breakdown of the overall simulated cattle population by production setting, while also reflecting the difficulties of depopulating large herd sizes seen in US feedlots.

### Economic model outcomes

Lost beef production due to clinical disease was small for any given quarter (< 1% per quarter), but losses aggregated over time as outbreak duration increased. The aggregate milk losses were larger than beef losses due to the assumed time to milk production recovery, however, the milk production losses represented a small proportion of the total milk produced in the United States (< 1%) (Table 7). Scenarios that incorporated the Texas panhandle resulted in minimal milk loss, with ~22% of iterations, originating in the Texas panhandle, resulting in disease spread to dairy production sites. The median (25th, 75th percentile) pounds of reduced milk production, due to disease, ranged from 3.33 million lbs. (41,206 lbs., 6.74 million lbs.) to 8.15 million lbs. (3.10 million lbs., 16.63 million lbs.) among scenarios. For context, annual US production of fluid milk was 217.6 billion lbs. in 2018 (44). Scenarios originating in California (scenarios, 1 vs. 4 and 3 vs. 6) resulted in statistically significant milk losses (*p* = 0.044) at the 5% level between scenarios 1 and 4 (depopulation at 28 vs. 14 days).

**Table 7:** Total economic outcomes from 2018 to 2022 associated with median (25th percentile, 75th percentile) disease outbreaks for: production losses of beef (thousands of pounds) and milk (millions of pounds) due to disease; on-farm government response cost (millions of dollars); and economic impact as measured by producer welfare (millions of dollars).

**Outcomes**	**Scenarios**					
	**1**	**2**	**3**	**4**	**5**	**6**
Milk losses (million pounds)	6.73 (3.10, 12.70)	7.64 (3.17, 14.21)	3.33 (0.04, 6.74)	7.63 (3.22, 16.99)	8.15 (3.20, 16.63)	3.18 (0.04, 5.54)
Beef Losses (thousands pounds)	196 (106,384)	402 (229, 579)	179 (178, 225)	246 (41, 450)	427 (237, 590)	179 (178, 228)
Cost of response (millions $)	$120 ($50, $189)	$76 ($70, $90)	$197 ($127, $276)	$136 ($44, $259)	$77 ($70, $100)	$228 ($138, $334)
Change in quarterly returns to beef cattle (millions $)	–$520 (–$405, –$640)	–$403 (–$397, –$510)	–$649 (–$402, –$522)	–$533 (–$405, –$634)	–$403 (–$397, –$402)	–$517 (–$405, –$638)
Chang in quarterly returns to dairy cattle and milk (millions $)	$7.48 ($6.82, $7.40)	$9.62 ($9.56, $12.07)	$11.58 ($7.05, $1.81)	$10.24 ($4.84, $4.22)	$9.73 ($9.58, $9.71)	$9.39 ($6.99, –$2.08)
Change in quarterly returns to capital and management on sales (millions $)	–$2,062 (–$1,614, –$2,508)	–$1,611 –$1,595, –$2,034)	–$2,532 (–$1,611, –$2,070)	–$2,097 (–$1,616, –$2,501)	–$1,611 (–$1,595, –$1,610)	–$2,056 (–$1,618, –$2,508)

The numbers in the table are the economic impacts for a variety of economic measures across a 4-year period (2018 to 2022) based on the median, 25th percentile and 75th percentile iterations of the disease spread outcomes as measured by head infected. For more details on economic outcome calculations, see the online Supplementary material.

Once culling was accounted for, longer-term impacts on beef production are more ambiguous with no clear-cut outcome across all scenarios. When evaluating the near term, cattle being fed for slaughter that were assumed to have experienced reduced ADG resulted in aggregate beef reductions ranging from 179,413 lbs. (177,841 lbs., 225,340 lbs.) to 426,915 lbs. (237,195 lbs., 589,874 lbs.) of beef never realized. Further complicating beef production impacts is the fact that many dairy bred steer calves and cull dairy cows are fed out for the beef market, meaning that both CA and TX have robust beef production. The estimated beef losses from scenarios involving both the Panhandle of Texas and California dairy production (3 and 6) were significantly larger when compared to the losses in the Panhandle scenarios alone (2 and 5) (*p* < 0.0001). However, when comparing the two depopulation strategies of 28- or 14- days, we did not find a significant difference in beef losses (*p* = 0.381).

The median cost of disease response in US dollars ranged from $76 million ($70 million, $93 million) to $230 million ($139 million, $339 million) (Table 7). For all scenarios except scenario 4 (CA, 14-day depopulation), indemnities paid on depopulated livestock represented the largest portion of outbreak response cost. When outbreaks originated in the Texas Panhandle indemnities accounted for up to 64% of the response cost. The median indemnity per outbreak ranged from $47 million ($12 million, $82 million) to $119 million ($71 million, $163 million). This was not an unexpected outcome considering that the region of interest was in a cattle dense area and fed beef cattle and lactating dairy cows are highly valued on a per head basis compared to other livestock types.

During the outbreak the second highest total on-farm response cost category was surveillance for all scenarios except scenario 4 (CA, 14-day depopulation), where it was the highest response cost category. Surveillance cost ranged from $8 million ($4 million, $13 million) to $62 million ($29 million, $106 million). With the reduced duration of depopulation to either 14 or 28 days, the cost of depopulation, disposal, and cleaning and disinfection did not represent a large portion of the total on-farm response cost. To illustrate this point, depopulation, disposal and cleaning and disinfection represented 16% of the estimated total on-farm response cost per head for each animal infected in this study; however, in these scenarios they represent only 12% and 10% of overall total on-farm response cost on average for 14 day scenarios and 28 day scenarios respectively. Spending shifts toward surveillance instead. As would be expected, the outbreaks with the lowest on-farm response cost resulted from the shortest and smallest outbreaks, which occurred in the Texas panhandle scenarios. Whereas, the highest on-farm response cost resulted from the longest and largest simulated outbreaks, which occurred in scenarios started in both CA and TX. Comparisons of total on-farm response cost between scenarios produced significantly different results (*p* < 0.05) for all scenarios based on start location and depopulation strategy except those originating in the Texas Panhandle.

Quarterly economic impacts were estimated over a 4-year period (2018 to 2021) and aggregated (Table 7). When comparing the median (25th, 75th) lost returns to capital and management (producer welfare), every scenario resulted in a statistically significant change from the pre-disease economic baseline (*p* < 0.05). Over the course of the 4-year period the markets did not recover to the pre-disease forecast of production returns for any of the 6 simulated scenarios. It has not been uncommon to see multi-year recovery periods in other countries after FMD outbreaks (45).

The pre-disease baseline quarterly returns to capital and management from sales of agricultural products averaged $23 billion. The median (25th, 75th percentile) outbreaks' average quarterly economic impacts for the lost returns to capital and management from sales ranged from $1,611 million ($1,595 million, $1,610 million) to $2,097 million ($1,618 million, $2,508 million), representing an average quarterly reduction of 7% to 11% in returns to producers and agribusinesses, across the agricultural sector. However, there was not a significant difference in the economic impacts, when evaluated at the median, 25th, or 75th percentile outbreaks. This could result from the influence of the export and consumer demand shocks on the economic impacts, which were similar across scenarios because of insignificant differences in epidemic duration.

In comparison, when the individual livestock industries were examined, the beef cattle sector's quarterly returns to beef cattle production were reduced in all 6 scenarios. Those reductions in returns ranged from $403 million ($397 million, $510 million) to $649 million ($402 million, $522 million). The swine and pork sector had the greatest loss to capital and management on sales of agricultural products at 61–64% while, the beef sector return reductions ranged from a 15–23%, when compared to the pre-disease baseline. The beef sector losses were the second highest industry specific component to the total (25–26%) followed by the red meat processing sector (14–17%). In contrast, the median (25th, 75th percentile) outbreaks for the dairy cattle and milk sector resulted in insignificant differences in producer returns compared to the pre-disease baseline; dairy sector economic impacts were moderated by the ability to export pasteurized and processed dairy products.

## Discussion

The potential use of vaccinate-to-live approaches for FMDV eradication is closely related to understanding the epidemiology and economic impacts of the carrier state; these considerations are highly specific to variations of specific outbreak contexts. In order to explore this question in a US production setting, we developed scenarios utilizing a vaccination-to-live strategy subsequent to depopulation of infected animals for a limited period (14 or 28 days). Overall, we found that economic production impacts varied across sectors, but were overshadowed by trade impacts associated with the estimated duration that carriers would be present in the population. Vaccinate-to-live may be attractive in terms of animal welfare, conservation of limited resources during response or for preserving valuable animal genetics. The long-term consequences on industry viability and farm and ranch longevity should be the subject of further research.

The model outcomes demonstrated that the epidemic size and durations estimated from a single index (dairy cattle) herd located in California (Figure 1) resulted in similar findings to that of a previous FMD modeling study conducted in the same state, as well as simulated outbreaks in European countries (46–49). Specifically, a review of FMD outbreaks conducted using real outbreak data in non-endemic countries reported that ~46% of epidemics had < 5 infected farms, 16% of epidemics had more than 150 infected farms, and another 16% of epidemics were extensive (>2,000 infected farms) (48). In the current study, outbreaks initiating from a feedlot in the Panhandle Region of Texas resulted in a smaller number of infected farms (median = 5), which is similar to the findings of the previously review (48). Another modeling study predicted smaller outbreaks when the index herd was beef cattle as was found in scenarios 2 and 5 of this study (46). Differences in movements associated with both direct and indirect contacts on dairy vs. feedlot operations likely drove this difference in outbreak size.

We found that, in most scenarios (iterations), early onset of vaccination reduced the epidemic size and depopulation burdens. For example, outbreaks with >100 infected farms were found in 31% of iterations in scenario 1 (vaccination onset on day 43), whereas only 23% of iterations reached this level when the vaccination was initiated on day 29 of simulation (Scenario 4). This is consistent with previous studies looking at FMD control in California and in Denmark (6, 49), which also found a similar epidemic duration and outbreak size. Though the epidemic size and length was reduced by early vaccination, the overall impact of vaccination on controlling an outbreak is influenced by several factors, such as available resources for vaccination and other control programs, compliance with movement restrictions and on farm biosecurity standards, and efficacy of the vaccines. As such, the use of vaccination must be considered in the context of the specific outbreak. In some settings, a particular vaccination strategy could result in overwhelming resource demands (humans, financial, and logistics) or result in extensive economic impacts (46). For example, the culling of vaccinated animals could increase the number of animals depopulated and be counterproductive considering the environmental impacts and resource allocations for carcass disposal and post-disposal activities. Additionally, there could be a shift in resource allocation and on-farm response costs as suggested in the economic model output, with indemnities paid out absorbing the largest portion of cost for all scenarios except scenario 4 where the outbreak was initiated on a dairy site in CA and depopulation was started at 14 days. In this scenario, surveillance absorbed the largest portion of cost. A vaccinate-to-live strategy could extend the trade ban period and result in the establishment of FMDV carrier animals (cattle, sheep and goats) in the population, which necessitates additional consideration for resource allocations for their management. As a result, the efficacy of vaccination in reducing outbreak size and duration should be balanced with an understanding of the additional resources and long-term implications of managing or disposing of vaccinated animals.

In this study, implementation of vaccinate-to-live strategy allowed up to 35% of infected cattle to remain in the population, and these cattle had the potential to become asymptomatic carriers of FMDV. Based on our modeling approach, the majority (57%) of non-depopulated, infected cattle transitioned into the carrier state after the first month of infection with the potential to remain in the population from 30 to 52 months post infection. Under such circumstances, the management of carrier animals would surely place an additional resource demand on response personnel. However, it is possible that this additional demand could be offset by reducing the resources required for depopulation and carcass disposal under a stamping-out or vaccinate-to-die strategy.

The results from the economic analysis suggested a reduction in overall on-farm response cost of 12% and 10% with the implementation of 14- or 28-day depopulation, respectively, from an estimated 16% under stamping-out. During a shorter and smaller epidemic (scenario 2 and 4), vaccination may not be beneficial as compared to stamping-out. Market impact analysis including the international and domestic trade consequences further affects the decisions regarding vaccination, depopulation and management of potential carrier animals (47). FMD is a disease that has the potential to cause considerable and lasting damage in export markets. In the case of outbreaks that are shorter and smaller, the economic damages from consumer avoidance and trade more than offset savings from reduced response costs. However, with the development of vaccines that can differentiate infected from vaccinated animals (DIVA), improved testing for carrier animals, and an improved understanding of the risks associated with carrier animals, an opportunity exists to refine trade embargo guidelines and regionalization agreements to account for alternative response strategies that may be needed in the event of resource constraints.

There was no conclusive impact on economic losses from the early onset of vaccination for on-farm response costs or economic returns as compared to later onset of vaccination. Government response costs were primarily associated with the indemnification of high value beef and dairy cattle and surveillance costs, including the costs of testing in vaccinated herds. Surveillance would be critically important to establish regionalization with key trade partners, and consequently limit trade impacts where possible. In this study, only Canada and Mexico were assumed to regionalize trade bans. Trade ban duration was linked to epidemic duration, based on the OIE standards, and epidemic duration was also used as the period of consumer avoidance. ISP results indicated that the only scenarios with significantly different simulated durations were scenarios 2 and 5, which had shorter durations than other outbreaks. Consequently, the trade embargo and consumer demand results were not greatly different except for scenarios 2 and 5. The economic losses in the Panhandle outbreaks were only 1% lower than other start locations in the 25th percentile but could range much higher (22%-36%) in larger simulated outbreaks. The greatest contributor to national economic loss was not the cost of managing carrier animals, but rather trade losses and consumer reaction; this coincides with studies of FMD vaccination in the US (50) and also with evidence from FMD outbreaks in other countries where vaccinate-to-live was practiced (51). As more scientific gaps are filled regarding FMDV persistence and transmission, there will be revision in FMD economic impact based upon how managers and consumers will respond to alternatives to stamping-out approaches. Further research is needed to address these gaps and refine analyses of vaccinate-to-live strategies given the potential of improved tracking and management carrier animals.

In executing this study, limited information was available on which to base assumptions of production losses in FMD recovered cattle, and these estimates could be improved by additional research on production losses in FMD-recovered herds. Although not explicitly examined in this study, it is possible that carrier animals would be removed from the herd more rapidly due to emergence of hoof deformity issues or other sequelae (14). A producer weighing the cost of monitoring and managing herd health in herds with carriers may not reap enough profit from recovered cows to keep those animals in production. Instead, those cows might be culled and replaced with new stock. Further, breeding stock producers with the highest potential gains associated with protecting genetic advances may also have the highest value associated with their brand and reputation. It may be more difficult to sell replacement animals out of vaccinated herds; however, there is no information on which to develop additional analyses regarding early culling due to reputation concerns at this time. Thus, the potential for livestock operations to accelerate removal of recovered livestock, or even go out of business, should be investigated more explicitly to fully understand the potential economic consequences of maintenance of carrier animals.

It is unknown at this time how US consumers would react to a vaccinate-to-live strategy without stamping out. Communication of scientific information on the safety of FMD-recovered animals living out their productive life and entering the US food chain would be crucial. It is also unknown how trade partners would react to FMD-recovered animals being allowed to continue production, given surveillance and tracing of recovered and vaccinated herds. However, even with relatively conservative trade embargo and consumer avoidance assumptions as compared to other studies (42, 50), beef markets did not recover to the pre-disease returns in the 4-year period examined. The uncertainty surrounding market recovery in the United States livestock industry, from a vaccinate-to-live without stamping-out strategy, could mean that there would be additional losses beyond the time period analyzed in this study. Improved understanding of the risks of carrier animals, along with higher potency vaccines and companion diagnostic tools, may contribute to shorter durations of risk-based trade embargoes in future outbreaks (8, 11). Further research that contributes to the understanding of FMD carrier risk may help align trade recovery guidelines, and perhaps reduce the economic burden associated with allowing recovered and vaccinated animals to live out a productive life.

The major caveat of this study is that the estimated outcomes are largely dependent on the input parameters and livestock demographics of the United States; therefore, extrapolation of these findings should be conservative. Further, it was necessary to assume that once animals entered the carrier phase, there was no transmission of FMDV; although this reflects the consensus of the published literature, it is also possible that low-level transmission does occur (11), which could have various downstream impacts on the findings herein. Similarly, simulations were conducted using serotype O-based transmission parameters, which may not reflect the full diversity of FMD viruses and transmission dynamics. Additionally, economic impacts were largely dependent on the parameters and baseline economic returns of the economic model, and the assumptions on trade and consumer avoidance. Both of these reactions may be influenced by risk perceptions associated with an individual outbreak (41, 52), and are very difficult to predict. Thus, these economic results should also be extrapolated cautiously.

## Conclusion

These results can be used to inform the consideration of vaccinate-to-live and controlled slaughter strategies for FMD outbreaks and the development of appropriate post-outbreak surveillance. Furthermore, this output will enable more detailed examination of the epidemiologic and economic implications of allowing convalescent cattle to survive and remain in production chains after FMD outbreaks in FMD-free regions. With the development of next generation DIVA vaccines, improved diagnostic tests to identify carriers, and an improved understanding of the risks associated with carrier animals, an opportunity exists to refine trade embargo guidelines and regionalization agreements to account for alternative response strategies to FMD outbreaks. It is envisioned that further improvement of vaccine and diagnostic technologies will contribute toward greater confidence in vaccinate-to-live strategies for FMD control.

## Data availability statement

The original contributions presented in the study are included in the article/Supplementary material, further inquiries can be directed to the corresponding author/s.

## Author contributions

SY and AD: conceptualization of research and study design. SY: model scenarios development, data analysis and visualization, and drafting of the manuscript. AH: economic assessment design and analysis. CS, AD, MB, KM-T, LH, and JA: contributions to writing, reviewing, and editing the manuscript. All authors contributed to the article and approved the submitted version.

## Funding

This project was funded through an interagency agreement between the USDA-Agricultural Research Service (ARS/USDA) and the Center for Epidemiology and Animal Health of the US Department of Agriculture (USDA), Animal and Plant Health Inspection Service. Additional funds came from ARS/USDA CRIS Project 1940-32000-061-00D. SY and KM-T were recipients of a Plum Island Animal Disease Center Research Participation program fellowship, administered by Oak Ridge institute for Science and Education (ORISE) through an interagency agreement with the US Department of Energy.

## Conflict of interest

The authors declare that the research was conducted in the absence of any commercial or financial relationships that could be construed as a potential conflict of interest.

## Publisher's note

All claims expressed in this article are solely those of the authors and do not necessarily represent those of their affiliated organizations, or those of the publisher, the editors and the reviewers. Any product that may be evaluated in this article, or claim that may be made by its manufacturer, is not guaranteed or endorsed by the publisher.
