# Supplementary material for: Epidemiologic and economic considerations regarding persistently infected cattle during vaccinate-to-live strategies for control of foot-and-mouth disease in FMD-free regions
Source: Front Vet Sci. 2022 Oct 20;9:1026592. doi: 10.3389/fvets.2022.1026592 (PMC9632437; doi:10.3389/fvets.2022.1026592)
Supplement: Supplementary file 1 [file Data_Sheet_1.pdf]

Epidemiologic and economic considerations regarding persistently infected cattle during vaccinate-to-live strategies for control of foot-and-mouth disease in FMD-free regions

Shankar Yadav[1,2,3], Amy H. Delgado[2*], Amy D. Hagerman[4], Miranda R. Bertram[1,3], Karla I. Moreno-Torres[1,2,3], Carolina Stenfeldt[1,5], Lindsey Holmstrom[2], Jonathan Arzt[1]

[1]Foreign Animal Disease Research Unit, Plum Island Animal Disease Center, Agricultural Research Service, United States Department of Agriculture, Greenport, NY, USA

[2]Center for Epidemiology and Animal Health, Animal and Plant Health Inspection Service, United States Department of Agriculture, Fort Collins, CO, USA

[3] Plum Island Animal Disease Center Research Participation Program, Oak Ridge Institute for Science and Education, Oak Ridge, TN, USA

[4]Department of Agricultural Economics, Oklahoma State University, Stillwater, OK, USA

[5]Department of Diagnostic Medicine/Pathobiology, Kansas State University, Manhattan, KS, USA

*Corresponding author:

Dr. Amy Delgado

Amy.H.Delgado@usda.gov

Supplementary Table S1: Descriptive statistics of livestock farms (bison, cattle, goat, sheep, swine, dealer) and their operation types included in the InterSpread Plus (ISP) model scenarios.

Farm type	Operation type	Herd size (range)	
		Small farm	Large farm
Cattle	Cow calf	1 to 199	200 to 7988
	Dairy	1 to 499	500 to 16416
	Feedlot	1 to 999	1000 to 100734
	Stockers	1 to 50528	
Bison	Bison	1 to 3623	
Goat	Goat	1 to 4837	
Sheep	Sheep	1 to 48160	
Swine	Enterprises	1 to 99	
	Farrow to feeder	100 to 979	1069 to 61039
	Farrow to finish	100 to 990	1016 to 167443
	Farrow to wean	100 to 996	1003 to 332446
	Grow to finisher	100 to 999	1000 to 422475
	Nursery	108 to 994	1001 to 58064
	Other	100 to 967	1008 to 174418
Dealer		1 to 130	

Supplementary Table (S2): Distributions of contact rates (frequency of contacts per day) assigned in the InterSpread Plus (ISP) model scenarios from origin farms and operations to markets.

**Operations**	**Contact rates**
Dairy (large)	Poisson (0.1216)
Swine small enterprises	Poisson (0.001)
Swine farrow to feeder	Poisson (0.004)
Swine farrow to wean	Poisson (0.004)
Swine nursery	Poisson (0.004)
Dealer	Poisson (0.222)
Swine farrow to finish	Poisson (0.001)
Swine grower to finish	Poisson (0.001)
Swine other	Poisson (0.001)
Cow calf (large) and stockers	Poisson (0.0089)
Bison and cow calf (small)	Poisson (0.0065)
Feedlot (large)	Poisson (0.0206)
Feedlot (small)	Poisson (0.0298)
Dairy (small)	Poisson (0.0374)
Dairy (large), goats, sheep, Swine enterprise	Constant (0)

Supplementary Table (S3): Movement distance (in kilometer (Km)) of cattle from the origin to destination farm and the probability movement in the different regions of the United States assigned in the InterSpread Plus model scenarios. A separate movement distance and probability of movement was assigned for large and small operation, whenever necessary.

Region	All	MW & PC		GL, NE, SE		All		All		All
Operations	Bison	Cow calf		Cow calf		Dairy		Feedlot		Stockers
Size	NA	Large	Small	Large	Small	Large	Small	Large	Small	NA
Distance (KM)	Probability of movement									
0	0	0	0	0	0	0	0	0	0	0
10	0.1	0.03	0.1	0.1	0.1	0	0	0	0	0.03
50	0.28	0.22	0.28	0.66	0.58	0.0005	0.0005	0.0005	0.01	0.22
100	0.17	0.5	0.17	0.18	0.17	0.062	0.062	0.062	0.99	0.12
200						0.2475	0.2475	0.2475		
250		0.12								0.09
500	0.45	0.09	0.45	0.06	0.15	0.69	0.69	0.69		0.5
1000		0.04								0.04

MW: Mid-West, PC: Pacific, GL: Great Lake, NE: North-east, SE: South-east region of the United States.

Supplementary Table (S4): Contact rates (movement per farm per day) and destination probabilities of direct swine movements within the U.S. assigned in the InterSpread Plus (ISP) model scenarios.

Movement type	Source farm types	Contact rate (movement /farm/day)	Destination farm type	Destination Probability
Breeding pigs (sow, gilt, boar)	Swine other (large) Swine other (small)	0.0413	Farrow to feeder (large)	0.0126
			Farrow to feeder (small)	0.067
			Farrow to finish (large)	0.2708
			Farrow to finish (small)	0.4239
			Farrow to wean (large)	0.1446
			Farrow to wean (small)	0.0611
			Small-scale enterprises	0.02
	Grower finisher (large) Grower finisher (small)	0.0015	Farrow to feeder (large)	0.0126
			Farrow to feeder (small)	0.067
			Farrow to finish (large)	0.2708
			Farrow to finish (small)	0.4239
			Farrow to wean (large)	0.1446
			Farrow to wean (small)	0.0611
			Small-scale enterprises	0.02
Weaned pigs		0.4068	Nursery (large)	0.8254

	Farrow to wean (large) Farrow to wean (small)		Nursery (small)	0.1546
			Small-scale enterprises	0.02
Feeder pigs	Farrow to finish (large) Farrow to finish (small)	0.0209	Grower finisher (large)	0.7397
			Grower finisher (small)	0.2603
	Farrow to feeder (large) Farrow to feeder (small)	0.1049	Grower finisher (large)	0.7297
			Grower finisher (small)	0.2503
			Small-scale enterprises	0.02
	Nursery (large) Nursery (small)	0.0868	Grower finisher (large)	0.7297
			Grower finisher (small)	0.2503
			Small-scale enterprises	0.02
Small-scale enterprises	Small-scale enterprises	0.0023	Small-scale enterprises	1
Market	Market	0.001	Farrow to feeder (large)	0.0267
			Farrow to feeder (small)	0.0535
			Farrow to wean (large and small)	0.0267
		0.004	Farrow to finish (large)	0.1337
			Farrow to finish (small)	0.2941
	Market	0.0077	Dealer	0.4386

Supplementary Table S5: Movement distance (in kilometer (Km)) of sheep and goat from the origin to destination farm and its probability movement assigned in the InterSpread Plus model scenarios.

Distance (km)	Probability of movement	
	Goat	Sheep
0	0	0
40	0.4151	0.2038
78	0.1698	0.0602
239	0.1227	0.3735
400	0.0943	0.0506
600	0.1981	0.3119

Supplementary Table S6: Movement distance (in kilometer (Km)) of dealers and to markets from the origin to destination farm and its probability movement assigned in the InterSpread Plus model scenarios.

Distance (km)	100	200	300	400	500	600	700	800	900
to market/ Dealer	0.83577	0.1129	0.02311	0.01195	0.01259	0.00299	0.00027	0.00037	0.00005

Supplementary Table S7: Movement distance (in kilometer (Km)) of indirect contacts (low risk and high risk) from the origin to destination farm and its probability movement assigned in the InterSpread Plus model scenarios.

Distance (KM)	High risk	Low risk
20	0.8184	0.9079
40	0.1205	0.053
60	0.0183	0.023
80	0.0231	0.0115
100	0.0034	
200	0.0093	0.0023
1000	0.007	0.0023

Supplementary Table S8: Median (25th, 75th percentile) number of infected and depopulated cow calf, dairy and feedlot and stocker cattle obtained from InterSpread model scenarios

**Scenario**	**Cow-calf (,000)**		**Dairy (,000)**		**Feedlots and stockers (,000)**	
	Infected	Depopulated	Infected	Depopulated	Infected	Depopulated
1	1.6 (0.06, 4.7)	0.07 (0.02, 0.2)	9.5 (4, 21)	7.5 (42, 13)	93 (2, 15)	65 (0.5, 123)
2	0.02 (0, 0.6)	0.15 (0, 0.56)	0 (0, 0)	0 (0, 0)	43 (42, 54)	43 (42, 53)
3	1.7 (0.6, 3.5)	1.1 (0.3, 3.3)	10 (4, 20)	9.5 (45, 17)	138 (70, 187)	108 (61, 166)
4	1.1 (0.1, 3.4)	0.3 (0.002, 0.8)	8.5 (4, 16)	6.3 (3.5, 10)	84 (11, 139)	56 (0.5, 94)
5	0.2 (0, 0.5)	0.009 (0, 0.3)	0 (0, 0)	0 (0, 0)	43 (42, 54)	43 (426, 44)
6	1.5 (0.4, 3.4)	0.5 (0.1, 1.2)	9.6 (4, 17)	7 (3.8, 11)	135(67, 179)	97 (52, 145)

Supplementary Table S9: Median (25th, 75th percentile) number of infected and depopulated livestock species included in the InterSpread Plus model scenarios.

**Scenarios**	**1**	**2**	**3**	**4**	**5**	**6**
Median (25th, 75th percentile) number of infected animals						
Bison	0 (0,8)	0 (0, 0)	0 (0, 8)	0 (0, 2)	0 (0, 0)	0 (0, 0)
Cattle (,000)	103 (10,172)	43 (42, 55)	148 (75, 205)	93 (17, 153)	43 (42, 54)	147 (74, 200)
Goat	94 (0, 925)	0 (0, 7)	72 (9, 609)	102 (9, 543)	0 (0, 9)	72 (8, 615)
Sheep	49 (0, 1900)	0 (0, 0)	46 (4, 852)	58 (0, 2293)	0 (0, 0)	65 (4, 1178)
Pig	7 (0, 549)	0 (0, 0)	9 (0, 768)	4 (0, 399)	0 (0, 0)	9 (0, 410)
Median (25th, 75th percentile) number of depopulated animals						
Bison	0 (0, 0)	0 (0, 0)	0 (0, 1)	0 (0, 0)	0 (0, 0)	0 (0, 0)
Cattle (,000)	79 (9, 136)	43 (42, 53)	118 (72, 182)	64 (7, 106)	43 (42, 47)	106 (60, 160)
Goat	18 (0, 112)	0 (0, 0)	37 (0, 125)	9 (0, 47)	0 (0, 0)	12 (0, 56)
Sheep	13 (0, 59)	0 (0, 0)	14 (0, 68)	4 (0, 39)	0 (0, 0)	0 (0, 0)
Pig	0 (0, 40)	0 (0, 0)	0 (0, 152)	0 (0, 0)	0 (0, 0)	0 (0, 6)

Supplementary Figure 1: Probability of infection transfer from various livestock farms and their operations after the onset of infection. (A) cattle operations (cow calf small and large, dairy small and large, feedlot small and large, and stockers) and (B) bison, goat, sheep, small swine enterprises, commercial swine farms, delays and markets.

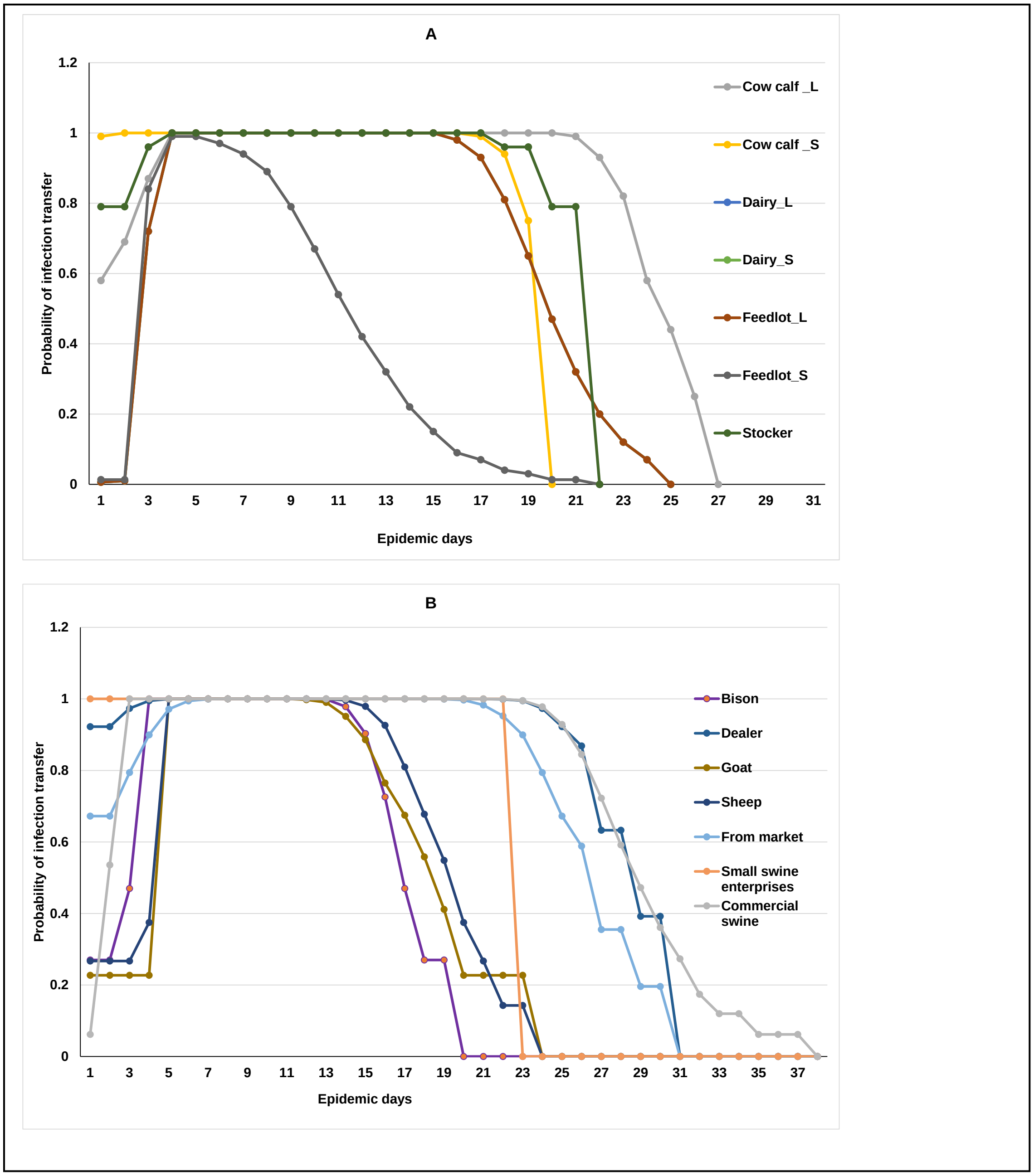

Supplementary Figure 2: Probability of infection transfer due to indirect (high risk and low risk) contacts.

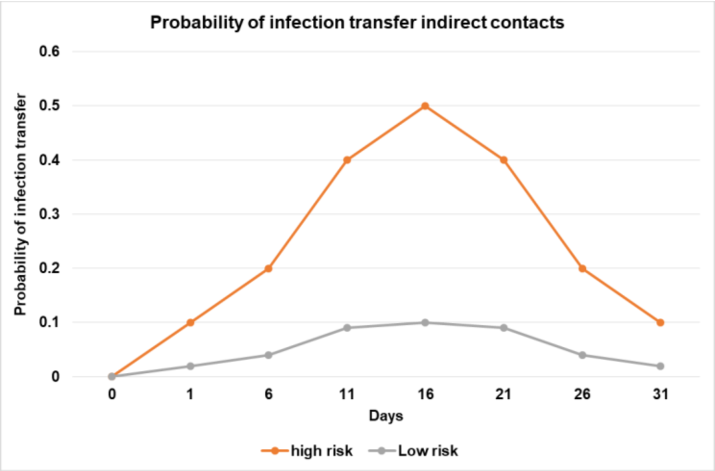

Supplementary Figure 3: Probability of transmission due to local spread and airborne mechanism (A) POT before detection (B) POT after detection but before depopulation, (C) POT after depopulation but before completion of the post-depopulation stage (e.g. cleaning, and sanitization), (D) Probability of infection transfer due to the airborne spread of foot-and-mouth disease, the line A and B represent the maximum and minimum POT incorporated in the model over the distance from the infected farms. Please consult the citation for a detailed description (1)

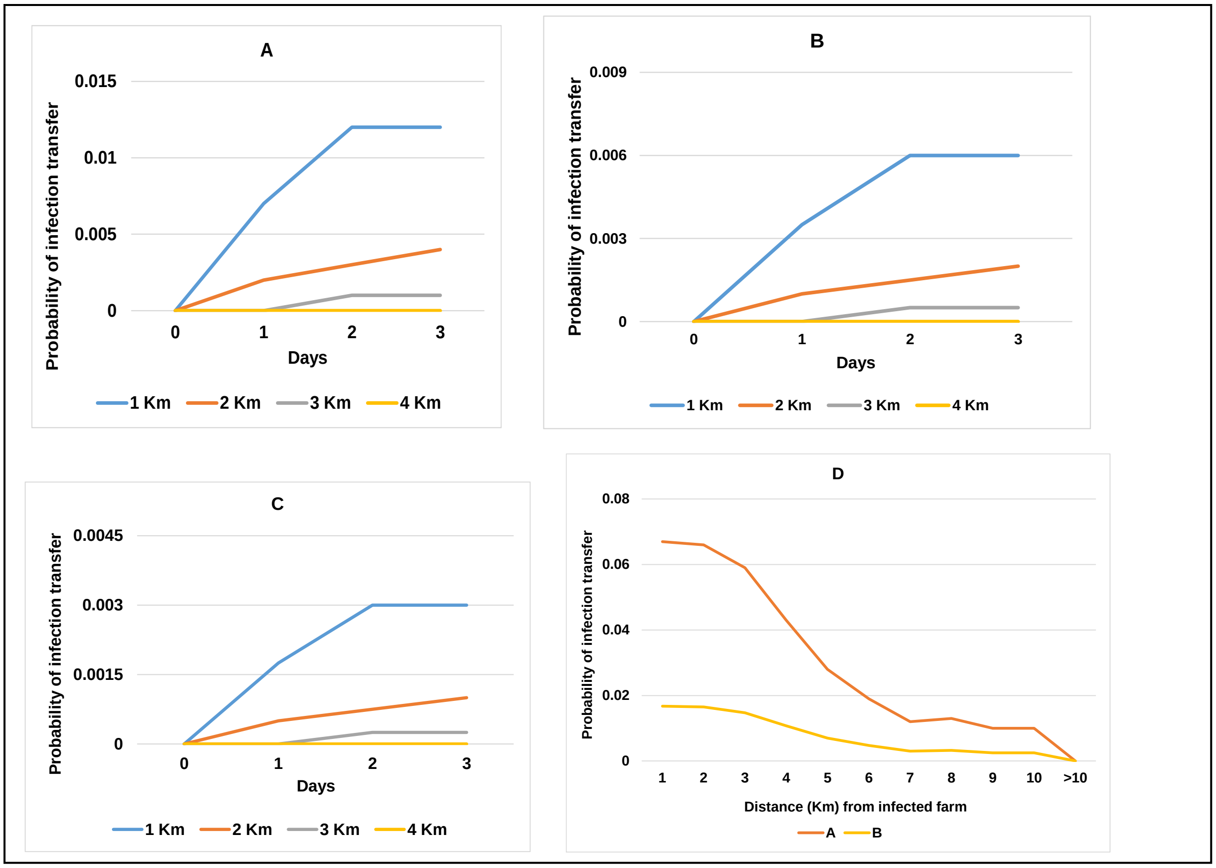

Supplementary figure 4: Probability of detection through passive surveillance incorporated in the InterSpread Plus Model for various livestock species after the onset of infection (1)

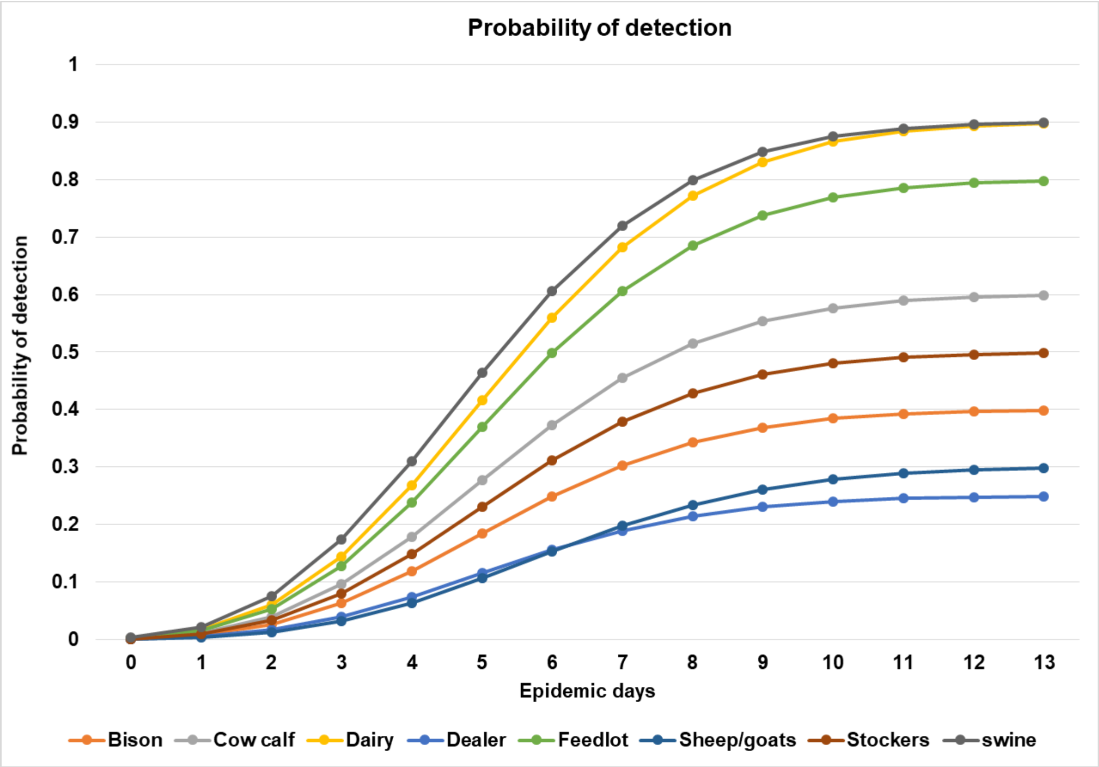

Cost of Response and Economic Model

Economic estimates of losses to producers and losses to the broader market, as well as costs of government response are outlined below.

Production Losses

Average daily weight gain (ADG) was assumed to be reduced by 1.4% (1) for each day of clinical disease. Although normal rates of ADG may be resumed at the end of clinical disease, beef cattle sent to slaughter after a typical 180 days on feed would be lighter than normal, reducing the supply of beef available to the market. Alternatively, feedlot owners could feed cattle longer, which increases the cost of production. Milk production in dairy cattle was assumed to reduce by 35% during clinical disease and recover at a uniform rate over a 7 month period post-infection (2).

Clinically infected beef and dairy cows were anticipated to abort calves at a rate of (10%) based on Doel (2003) (3) but no evidence was available in the literature to include a long term reduction in fecundity for beef or dairy cows (4). It is difficult to say whether dairy producers would continue to milk lactating dairy cows that aborted or dry them off and subsequently cull them. That choice may depend largely on the market conditions and availability of replacements. Since calving results in the onset of lactation, dairy cows that aborted were assumed to be culled upon recovery/drying out when animal movements resume. No additional culling in beef cows was assumed due to abortions since there is no evidence of reduced fecundity post-recovery. The death rate in unweaned calves was assumed to be 2.8% (5).

On-Farm Government Response Costs

Total on-farm costs of disease response included surveillance, depopulation and indemnification of depopulated animals, disposal of carcasses and potentially contaminated materials, cleaning and disinfection of facilities, and vaccination. Response costs were estimated in US dollars per head by production type. Costs for supplies and equipment for depopulation, disposal, decontamination, and

cleaning and disinfection of facilities were based on commercial prices from vet supply firms in 2017, with the exception of surveillance. USDA employee labor cost was based on the GS scale available online for 2018. Depopulation cost was a per animal and included labor hours by VS personnel, supplies and equipment for a tranquilizer to render animals unconscious followed by euthanasia solution for bison ($125/head), cattle ($57/head), swine ($18/head), and small ruminants ($31/head). The need for additional equipment to process animals, by animal type, for depopulation was included in the cost. Disposal cost included labor, supplies and equipment but varied based on animal type and the geographic location where that animal type is common. For example, burial is unlikely for dairy cattle, given rules in dairy intensive west coast regions associated with burial. The disposal cost was based on using a landfill for cattle in feedlots ($143/head), on-site burial for breeding beef cattle herds ($34/head) and bison ($37/head), rendering for dairy cattle ($61/head) and swine ($11/head), and composting for small ruminants ($32/head). Cleaning and disinfection costs included labor, equipment and supplies, and detergents and disinfectants for cattle ($113/head), swine ($14/head) and small ruminants ($99/head). The variation in cost by cattle production type, in particular, is likely to be high; the cost estimate used here were intended to be an average cleaning and disinfection cost across operation types within a species.

Indemnity for depopulated livestock was based on USDA-AMS data for 2018, by livestock type, multiplied by the average weight of each animal type in the herd to get a weighted average indemnity value across the herd. Livestock valuation for indemnification was assumed to cost $86/animal regardless of animal type for the labor to enter livestock in pre-determined livestock indemnity calculator formulas and generate the necessary forms.

Vaccination costs included labor, equipment for administering the vaccine, and supplies including the vaccine, syringes, needles, biohazard disposal containers for used supplies, and ear tags for tracking vaccinated animals. Costs were estimated for cattle ($38/head), swine ($26/head), and small ruminants ($44/head).

Surveillance cost during the disease outbreak included a per farm component for labor and equipment to take samples, as per animal sampled cost for test kits and a per sample cost for laboratory testing. These were aggregated and divided by the average number of head per farm for a cost for cattle and bison of ($69/head), swine ($55/head), and small ruminants ($65/head). The US FMD Response Plan, in describing a vaccinate-to-live without stamping out response, indicates that "DIVA testing may be necessary for movement between zones, interstate commerce, and international trade." (6). To establish an "FMD Free Zone where Vaccination is Practiced" requires surveillance for FMD circulation. However, the Response Plan does not outline an exact surveillance strategy. The exact strategy would be dependent on the situation and a variety of considerations in the outbreak characteristics and resources available.

Market Demand Loss Estimates

Market losses originate from 3 sources: production losses from the disease and depopulation, trade embargoes (international demand), domestic consumer avoidance (domestic demand). First, sanitary restrictions on trade would be expected to result in trade losses for beef, pork and limited dairy products. National trade bans were initiated at the onset of disease for all susceptible livestock species and animal products from those species that had not been processed in a way that would kill the virus (e.g. dried milk powder). The trade bans were assumed to remain in place for the duration of the outbreak, due to the vaccinate-to-live without stamp-out response. The OIE Terrestrial Animal Health Code, Article 8.5.9, requires a waiting period of "six months after the last case or the last vaccination (according to the event that occurs the latest) where a stamping-out policy, emergency vaccination was not followed by the slaughtering of all vaccinated animals, and serological surveillance are applied in accordance with Articles 8.5.42. to 8.5.47. and Article 8.5.49., provided that a serological survey based on the detection of antibodies to non-structural proteins of FMDV demonstrates the absence of infection in the remaining vaccinated population." Based on these guidelines, national trade embargoes were assumed to remain in place until 6 months after the last carrier animal was removed or ceased to be a viable carrier via serological sampling for all trading partners except Mexico and Canada. Close trade relations with those countries may result in

regionalized trade embargoes, and consequently the ability to ship product from animals that were originated and processed in disease free areas. Regionalization was shown to reduce trade consequences in HPAI during the 2014-2015 outbreak by Thompson et al. (2017) (7) and has been shown in modeling studies to reduce potential losses for FMD in Paarlberg et al. (2006) (8).

Although the US exports a portion of meat and animal production to international partners, the majority of US animal agriculture production is consumed domestically--more than half of US produced pork, beef and dairy products is consumed domestically. Domestic demand has been found to decline in response to an animal health events in the US, but have historically remained relatively small (7). Trade embargoes create a surplus of affects products for domestic markets, which can reduce prices if production losses are relatively smaller than trade reductions. Resulting declines in meat prices can lead some consumers to substitute more beef or pork into diets, offsetting the avoidance of beef and pork by other consumers with unfounded food safety concerns regarding FMD in domestic herds. Mu et al. (2015) found that US consumers adjusted to information on food safety relatively quickly. Specifically, Mu et al. found that bovine spongiform encephalopathy (BSE) announcements in 2003, 2006 and 2010 resulted in declines in beef demand after each event but were statistically insignificant and small at 0.42%. Analysis supports prior literature that indicates that US consumers consider beef and other meats (except pork) to be necessities (7). Based Mu et al.’s findings, a shallow, negative shock (-0.5%) was imposed to US beef and pork demand that was sustained through the outbreak. Recovery was allowed to occur quickly afterward.

US Partial Equilibrium Model

The impact of production losses, depopulation and death losses, trade embargoes and domestic consumption losses on markets were estimated using the United States Partial Equilibrium Model (USPEM) (8). This model is a national price-endogenous economic model that estimates changes in market prices and economic welfare in calendar quarter time steps. USPEM has 33 livestock categories, including 11 final agricultural industries (e.g. beef, pork, lamb) as well as primary and intermediate inputs. The changes in prices and quantities in the model were primarily driven by revenue shares for each agricultural sector,

elasticities, the feed-balance calculations that tie the livestock and grain sectors together, and the exogenous shocks that the user imposes. Each final product was defined by an initial equilibrium that included a price, quantity and elasticities that define the relationship of price and quantity as well as the prices between each of the 11 products. Production losses and demand shocks, as described above, were imposed on the model as exogenous shocks. Changes in quantity supplied and quantity consumed that resulted from price changes were solved endogenously in the model. Output includes market prices and producer welfare, which is defined as the difference between the schedule of prices at which producers are willing and able to supply a good in varying quantities supplied, and the price they actually realize in the market for those quantities supplied. It is different from profit in that producer welfare accounts for fixed, or sunk, costs of production.

References

1. USDA. Parameters Used to Simulate the Spread of FMD in Texas Using the North American Animal Disease Spread Model (NAADSM) for use in FMD Response Workforce Requirement Estimates. (2013).
2. Lyons NA, Alexander N, Stärk KD, Dulu TD, Sumption KJ, James AD, Rushton J, Fine PE. Impact of foot-and-mouth disease on milk production on a large-scale dairy farm in Kenya. *Preventive veterinary medicine* (2015) 120:177–186.
3. Ranjan R, Biswal JK, Subramaniam S, Singh KP, Stenfeldt C, Rodriguez LL, Pattnaik B, Arzt J. Foot-and-mouth disease virus-associated abortion and vertical transmission following acute infection in cattle under natural conditions. *PloS one* (2016) 11:
4. Doel T. FMD vaccines. *Virus Research* (2003) 91:81–99.
5. Rufael T, Catley A, Bogale A, Sahle M, Shiferaw Y. Foot and mouth disease in the Borana pastoral system, southern Ethiopia and implications for livelihoods and international trade. *Tropical animal health and production* (2008) 40:29–38.
6. USDA APHIS FMD Response Plan: The Red Book. (2012)
7. Mu JE, McCarl BA, Hagerman A, Bessler D. Impacts of bovine spongiform encephalopathy and avian influenza on US meat demand. *Journal of Integrative Agriculture* (2015) 14:1130–1141.
8. Paarlberg PL, Lee JG, Seitzinger AH. Potential revenue impact of an outbreak of foot-and-mouth disease in the United States. *Journal of the American Veterinary Medical Association* (2002) 220:988–992.
